# Strongyloidiasis in primary care: Challenges in the diagnosis and management of a neglected infection^✰^

**DOI:** 10.1016/j.clinsp.2026.101150

**Published:** 2026-09-21

**Authors:** Guilherme França da Silva, Pablo Lorran Pereira Santos, Paula Yuri Sugishita Kanikadan, Ronaldo Cesar Borges Gryschek, Fabiana Martins de Paula, Marcelo Andreetta Corral

**Affiliations:** a Faculdade de Medicina, Universidade Santo Amaro (UNISA), São Paulo, SP, Brazil; b Laboratório de Investigação Médica (LIM-06), Hospital das Clínicas da Faculdade de Medicina da Universidade de São Paulo, São Paulo, SP, Brazil; c Departamento de Patologia Básica, Universidade Federal do Paraná, Curitiba, PR, Brazil

Strongyloidiasis is a Soil-Transmitted Helminthiasis (STH) caused by the nematode *Strongyloides stercoralis*, with a global prevalence estimated at 600 million people, despite significant underreporting.^1^^,^^2^ The disease is intrinsically linked to contexts of socioeconomic vulnerability and a lack of basic sanitation, particularly in tropical and subtropical regions, characterizing it as a Neglected Tropical Disease.^1^^,^^3^ In Brazil, the infection remains an expression of historical inequities in rural areas and urban peripheries.^4^^,^^5^

The primary challenge for disease control is its sanitary invisibility, fueled by low diagnostic sensitivity and systematic underreporting.^1^^,^^2^ Biologically, the parasite is distinguished from other STHs by its capacity for autoinfection, a process allowing the completion of the biological cycle within the host itself.^6^ This phenomenon ensures a potentially lifelong and silent chronic infection, which may persist for decades without re-exposure to the environment. Furthermore, the clinical presentation of strongyloidiasis in immunocompetent individuals is predominantly asymptomatic or characterized by nonspecific symptoms, such as intermittent abdominal pain, episodic diarrhea, and dry cough.^6^, ^7^, ^8^

Given this clinical non-specificity, which frequently leads to misdiagnoses of functional gastritis or irritable bowel syndrome, it is essential, when suspicion arises in Primary Health Care (PHC), to utilize the laboratory marker of persistent peripheral eosinophilia, which must always prompt the investigation of helminthiases in endemic areas.^2^^,^^7^ When escalating diagnostic investigation, the interpretation of findings suggestive of strongyloidiasis must be conducted with caution, as the field still lacks tools with robust performance and adequate validation.

The accuracy of available methods is heterogeneous and often overestimated in the literature, especially in studies conducted in high-parasitic load contexts, which limits their extrapolation to low-endemicity scenarios or low-intensity chronic infections. Conventional parasitological methods remain widely used despite their recognized low sensitivity, particularly due to intermittent larval shedding, which compromises their utility as a rule-out diagnostic tool.^9^^,^^10^ While methods such as Baermann-Moraes and agar plate culture are more effective, they are rarely available in routine PHC.^2^^,^^10^

Regarding serology, although widely promoted as a more sensitive alternative, its applicability is restricted by specificity issues arising from cross-reactivity with other helminthiases, a bias particularly relevant in tropical regions where polyparasitism is frequent. This scenario can result in overdiagnosis and subsequent overtreatment, especially when serology is employed in isolation. Moreover, the prolonged persistence of antibodies post-cure limits its utility in therapeutic monitoring, hindering the distinction between active infection and prior exposure.^11^ Such weaknesses are even more evident in the PHC context, where structural constraints, the absence of high-accuracy methods, and limitations in longitudinal follow-up capacity often render the diagnosis presumptive. In this setting, the reliance on clinico-epidemiological criteria and the adoption of empirical strategies, while pragmatic, expose the tension between clinical practice and the fragility of available evidence, highlighting the urgent need for the development and validation of more precise and accessible diagnostic tools.

In scenarios where access to high-sensitivity tests is limited, empirical therapy prior to immunosuppression is advocated as a cost-effective strategy to prevent fatal hyperinfection in individuals initiating corticosteroids or biological agents.^8^^,^^12^ Post-treatment monitoring presents a logistical challenge, as parasitological cure is difficult to verify via conventional methods and serology exhibits a slow decline in antibody titers.^10^^,^^11^ Thus, clinical follow-up and continuous epidemiological risk assessment are essential in PHC.

Strongyloidiasis is a deeply territorialized disease, with exposure patterns shaped by precarious housing and labor conditions. PHC, through the Family Health Strategy (FHS), is the privileged instance to address these determinants.^3^ As part of the social determinants, inadequate sanitation and poverty sustain the disease within the territory. Primary prevention requires intersectorality, as hygiene guidance is insufficient without robust sanitation policies and housing infrastructure.^3^^,^^13^

Based on this premise, home visits allow the *in loco* observation of risks that clinical consultation cannot reach, such as open-air sewage, absence of footwear, and direct contact with moist soil, expanding the knowledge regarding the territory.^14^ The Community Health Agent plays a strategic role by identifying vulnerable groups and patients using immunosuppressants in risk areas, mediating the link between the community and active surveillance.^15^^,^^16^ Consequently, permanent education for health professionals must be implemented to increase the index of clinical suspicion. For the community, Freirean-based popular education promotes autonomy and contextualized protective practices.^17^

It is necessary to emphasize that early identification is critical due to the risk of hyperinfection syndrome, especially in immunosuppression contexts.^8^^,^^18^ The use of corticosteroids, even in moderate doses or during acute treatments, as observed during the COVID-19 pandemic, can accelerate the autoinfection cycle and cause massive larval dissemination with high mortality.^8^^,^^19^ Other risk factors include hematological malignancies and HTLV-1 co-infection, which compromise the antiparasitic immune response.^5^^,^^6^ In cases of uncomplicated strongyloidiasis, the drug of choice is ivermectin (200 µg/kg in a single dose, which may be repeated in 7 to 14 days), which demonstrates therapeutic superiority and better tolerability compared to albendazole.^13^^,^^18^^,^^20^ In PHC, management must be active even in asymptomatic patients from endemic areas to interrupt the autoinfection cycle.^13^

The integration of strongyloidiasis into the PHC agenda is a political-sanitary project grounded in equity.^21^ The invisibility of the disease in notification systems compromises planning and reflects the neglect of conditions affecting marginalized populations.^3^^,^^22^

Confronting strongyloidiasis within the Brazilian Unified National Health System (SUS) must be technical (early diagnosis), ethical (care for the vulnerable), and political (strengthening the SUS). Integrating this condition into the Primary Care routine is not merely a protocol adjustment, but a reaffirmation of the public system’s commitment to sanitary justice and the prevention of avoidable deaths.

## Declaration of generative AI and AI-assisted technologies

The authors declare that they did not use generative artificial intelligence tools or AI-assisted technologies in the writing of this manuscript, except for limited use in checking references. All scientific content, interpretations, and conclusions are the sole responsibility of the authors.

## Authors’ contributions

Guilherme França da Silva and Pablo Lorran Pereira Santos participated in the conception of the manuscript, literature search, reading and analysis of the collected material, and final writing of the article. Paula Yuri Sugishita Kanikadan participated in the conception of the manuscript, reading and analysis of the collected material. Ronaldo Cesar Borges Gryschek, Fabiana Martins de Paula and Marcelo Andreetta Corral participated in the conception of the manuscript, reading and analysis of the collected material, supervision and final revision of the writing.

## Funding

None.

## Data availability

Data available within the manuscript.

## Declaration of competing interest

The authors declare no conflicts of interest.
